# Posterior Ankle Impingement Syndrome Caused by Nonunion of Isolated Posterior Malleolar Fracture

**DOI:** 10.7759/cureus.79208

**Published:** 2025-02-18

**Authors:** Takao Minami, Yasuhiro Nakane, Norihiko Nakaima, Munehito Yoshida

**Affiliations:** 1 Orthopaedic Surgery, Sumiya Orthopaedic Hospital, Wakayama, JPN; 2 Orthopaedic Surgery, Nakaima Orthopaedic Hospital, Iwade, JPN

**Keywords:** ankle sprain, arthroscopic surgery, isolated posterior malleolar fracture, nonunion, posterior ankle impingement

## Abstract

Isolated posterior malleolar fracture is a rare condition, and this fracture is usually overlooked and diagnosed as an ankle sprain owing to a lack of awareness and difficulty in diagnosis. Posterior ankle impingement syndrome (PAIS) is relatively common in athletes and is usually caused by trauma or overuse. The impingement occurs due to repeated compression of a bony or soft tissue. Here, we report a rare case of a 17-year-old rugby player presenting with posterior ankle impingement caused by nonunion of an isolated posterior malleolar fracture. This patient was treated using a posterior ankle arthroscopic excision. At 16 weeks postoperatively, his ankle and hallux pain disappeared, and he returned to sports activities with no morbidities related to the surgical procedure. Based on a computed tomography scan, the bone fragment causing the impingement was resected, and the union of the residual posterior malleolar fragment was verified.

## Introduction

While posterior malleolar fracture is usually complicated with other ankle fractures, “isolated” posterior malleolar fracture is a rare condition [1,2]. Because isolated posterior malleolar fracture can easily be missed on plain radiographs and lacks specific symptoms, it is usually overlooked and diagnosed as an ankle sprain [3]. Although previous studies have described that almost all cases of isolated posterior malleolar fracture had a good outcome [1], Comat described that delayed diagnosis cases were associated with prolonged incapacity and more severe physical sequelae [4]. Because there are few reports about this fracture, the prognosis of this fracture is unclear.

Posterior ankle impingement syndrome (PAIS) is a clinical disorder characterized by posterior ankle pain that is caused or aggravated by forced plantar flexion. The syndrome is relatively common in athletes and is usually caused by trauma or overuse [5]. Posterior ankle impingement occurs due to various pathologies, such as os trigonum, hypertrophic posterior talar process or Stieda process, Shepherd’s fracture, thickened posterior ankle joint capsule, torn posterior inferior tibiofibular ligament, posttraumatic soft tissue scar, loose body, and anomalous muscles [5,6-8]. Because of the close anatomic orientation of the flexor hallucis longus (FHL) and posterior talar process, FHL tendinopathy is often associated with PAIS [9,10]. Although conservative therapy is effective in many cases, surgery may be required in refractory cases. Traditionally, open surgical procedures have been performed with good results. Recently, arthroscopic procedures have been widely accepted because of earlier return to sports activities and lower complication rates [9,11,12]. This report describes a rare case of posterior ankle impingement caused by an untreated nonunion of an isolated posterior malleolar fracture.

## Case presentation

A 17-year-old high school rugby player presented with left posterior ankle and hallux pain. He sprained his left ankle during a rugby game two weeks earlier. He could play rugby after the injury, but he still complained of posterior ankle and hallux pain. He had a history of a serious ankle sprain on the same side in the previous year. At that time, he was limping and received treatment for swelling and pain in the ankle joint; no fracture was detected, and he was diagnosed and treated with an ankle sprain. When he visited our outpatient clinic, he was able to walk comfortably without limping. Physical examination revealed mild swelling and localized tenderness over the posteromedial aspect of the ankle. Although the movement of the ankle joint and the hallux was smooth without a limited range of motion, posterior ankle pain and hallux pain were aggravated by forced plantar flexion of the ankle joint and plantar flexion of the hallux with counter resistance. Also, he could not stand on tiptoes because of these symptoms. Anterior drawer test was negative, and stress testing results for anterior, eversion, and inversion were similar on both sides.

**Figure 1:**
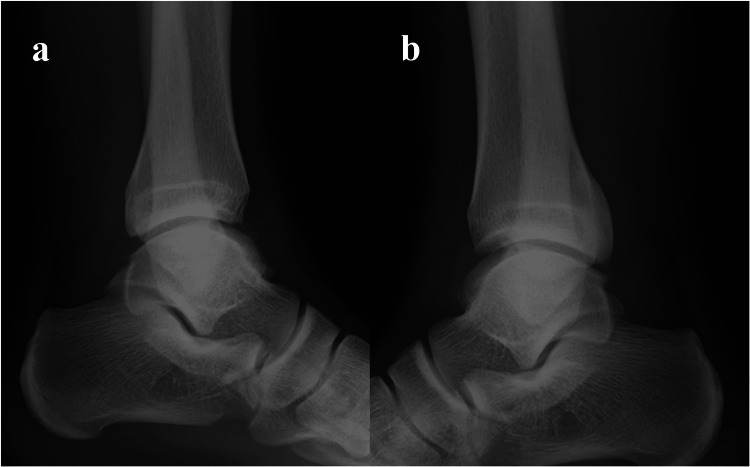
Lateral ankle radiography at the initial visit (a) Unaffected side. (b) Affected side

**Figure 2:**
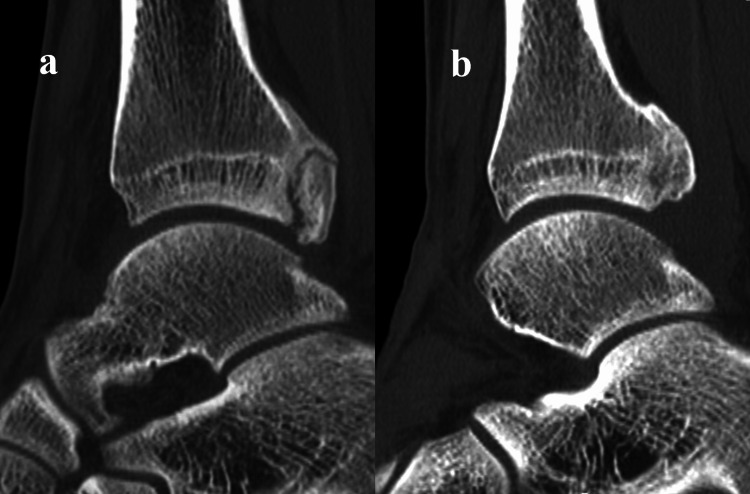
Sagittal reformat of a computed tomography (CT) scan (a) Preoperative image demonstrates nonunion with malformed callus of posterior malleolar fracture. (b) Postoperative image demonstrates union of the residual fragment

**Figure 3:**
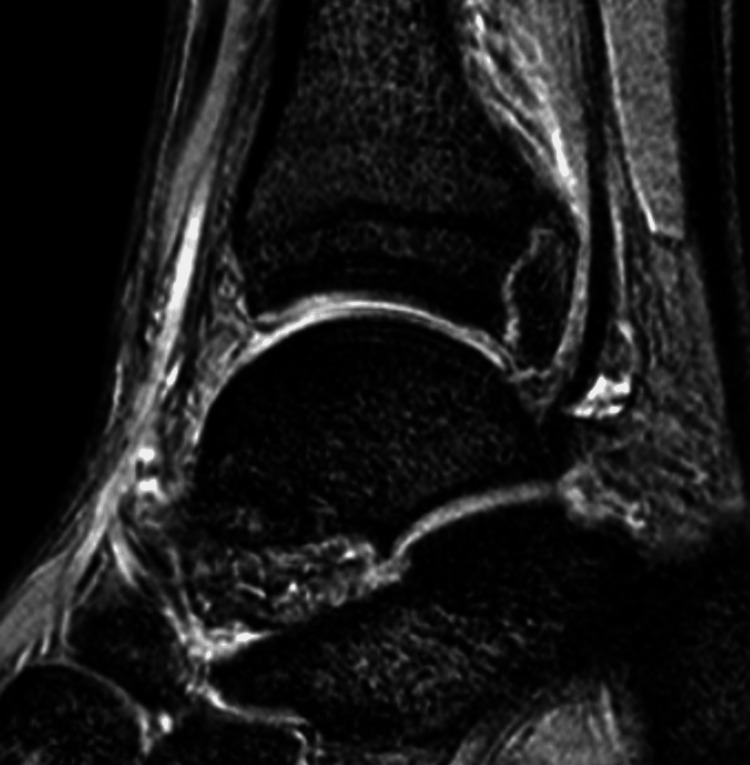
Preoperative T2-weighted sagittal magnetic resonance FHL: flexor hallucis longus Imaging shows tenosynovitis around FHL

Radiographs showed no definite fracture, but the posterior malleolar surface was not similar to the unaffected side (Figure 1). A computed tomography (CT) scan revealed nonunion of a posterior malleolar fracture, with a malformed callus (Figure 2a). The nonunion fragment was relatively large, with a callus and irregularities on the articular surface. Articular involvement was 7% of the tibial plafond, which correlates to type 3 using the Haraguchi classification [13]. Magnetic resonance imaging (MRI) showed edematous changes in the bone fragment and increased intensity surrounding the FHL tendon (Figure 3). A diagnosis of posterior ankle impingement caused by nonunion of isolated posterior malleolar fracture and FHL tenosynovitis was made. A trial of conservative treatment included physical therapy, medical treatment, and local corticosteroid injections implemented for six months. These treatments were effective to some degree, and he was able to continue competing while undergoing conservative treatment; however, symptoms remained. Ultimately, the pain worsened, so arthroscopic surgery was performed to remove the pathology causing the impingement.　

**Figure 4:**
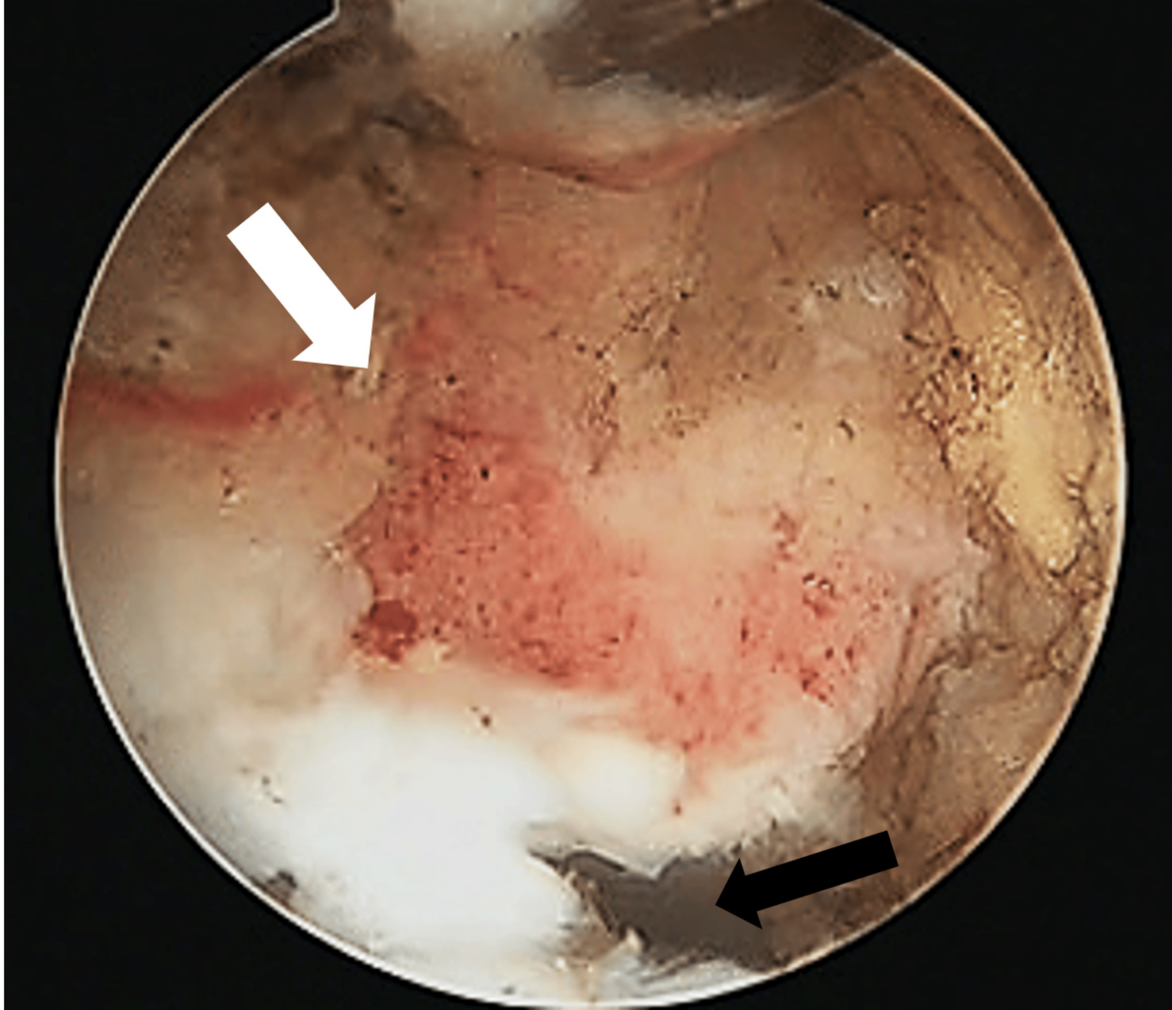
Arthroscopic viewing Resection of posterior malleolar bone fragment causing impingement, using a chisel. The white arrow indicates the bone fragment. The black arrow indicates the ankle joint

The surgery was performed in the prone position under general anesthesia. We used posterolateral and accessory posterolateral portals that were originally described by Horibe [14]. After the removal of the fibro-fatty tissue of the posterior ankle joint space, the FHL tendon and inflammatory synovia were identified. The tendon was mobilized smoothly by removing the synovia. A release of the ankle joint by posterior capsulotomy was performed. A posterior malleolar fragment that impinged on the posterior talar process during forced plantar flexion was resected using a chisel (Figure 4). The residual bony fragment was not unstable. Postoperatively, splint fixation was performed for a week. After that, weight-bearing as tolerated and range of motion exercises were permitted. By the sixth week, the patient was able to run. Preoperative posterior ankle pain and hallux pain were no longer present, and the patient returned to sports activities 16 weeks after surgery. No morbidities related to the surgical procedure and no laxity were observed. On a follow-up CT scan, the union of the posterior malleolar fragment was verified, and the distal part of the bone fragment was partially resected (Figure 2b). A radiograph at the final follow-up 12 months postoperatively showed no regrowth of the posterior malleolar fragment and definite degenerative changes. The Preoperative American Orthopedic Foot and Ankle Society (AOFAS) score was 74, whereas AOFAS score at the time of the final follow-up increased to 100, and the visual analog scale reduced from 7/10 to 1/10. Informed written consent was obtained from the patient.

## Discussion

This case illustrates that overlooked and untreated isolated posterior malleolar fracture induced PAIS. Many pathologies have been described as causes of PAIS. In a recent review of PAIS, os trigonum and Stieda process have been mentioned as common causes of bony impingement, whereas FHL pathology is a common cause of soft tissue impingement [5,6-8]. Jourdel reported cases of PAIS caused by a malformed callus of the posterior malleolus [6], but such cases were rare. Although conservative therapy is the first choice, a surgical approach is required when it is ineffective. In recent years, arthroscopic surgery has gained popularity because of less scarring, earlier return to activities, and lower complication rates.

Isolated posterior malleolar fracture is a rare condition that accounts for 1%-4% of all ankle fractures [1,2]. The mechanism of this fracture is axial compression force on the posterior malleolus from the talus under plantar flexion [15]. This form of fracture is usually overlooked and diagnosed as an ankle sprain owing to a lack of awareness and difficulty in diagnosis [3]. Previous studies have described difficulty in diagnosis, as the fracture provides poor specific symptoms on clinical examination, and can easily be missed on plain radiographs [4,15]. On the other hand, Ebraheim suggested a radiograph in 50° external rotation to assess the posterior malleolus [16]. Therefore, if this fracture is suspected, radiographs should include a view in 50° external rotation. There are controversies about the treatment of posterior malleolar fracture. Many studies agree that small fragments can be treated conservatively, while larger fractures involving more than 25% of the articular surface should be fixed to avoid instability and degenerative changes [17,18]. In contrast, biomechanical studies have described that posterior malleolar fragments lead to increased osteoarthritic changes even when fragments are small [19]. However, these studies considered posterior malleolar fractures complicated by other ankle fractures. Therefore, the concept of treating “isolated” malleolar fractures may be different as per these biomechanical studies. There are few reports about the prognosis of isolated fractures. Donken described that almost all cases of isolated posterior malleolar fracture resulted in good clinical and radiological outcomes on long-term follow-up [1]. Ozler reported that there was no instability and degenerative change at the first-year follow-up in seven patients, including one with nonunion [3]. However, Comat described that 75% of cases with delayed diagnosis were associated with prolonged incapacity and more severe physical sequelae, such as cracking sensation, pain, and stiffness in spite of the fractures not being displaced [4].

In our case, a posterior malleolar fracture might have occurred when a previous serious injury was diagnosed as an ankle sprain. Impingement that occurred in the posterior malleolar fragment with a malformed callus and the posterior talar process triggered an acute ankle injury. Micromotion of the enlarged and deformed bone fragment induced tenosynovitis around the FHL. Only 7% of the articular surface was involved, and the major symptoms were induced by impingement. Therefore, we performed arthroscopic surgery to resect the cause of impingement. After surgery, the union of the residual fragment was verified because of the disappearance of micromotion.

## Conclusions

We present a rare case of posterior ankle impingement caused by nonunion of an isolated posterior malleolar fracture. The patient returned to full function after arthroscopic surgery. To prevent unexpected sequelae and manage appropriately with early diagnosis, physicians need to suspect this fracture from mechanism. If this fracture is suspected, radiographs should include an external rotation view in addition to the standard two-view. In the case of persistent posterior ankle pain, reexamination is recommended.
